# Whole genome duplication is an early event leading to aneuploidy in *IDH*-wild type glioblastoma

**DOI:** 10.18632/oncotarget.26330

**Published:** 2018-11-13

**Authors:** Blandine Boisselier, Frédéric Dugay, Marc-Antoine Belaud-Rotureau, Anne Coutolleau, Emmanuel Garcion, Philippe Menei, Philippe Guardiola, Audrey Rousseau

**Affiliations:** ^1^ Département de Pathologie Cellulaire et Tissulaire, CHU Angers, Angers, France; ^2^ CRCINA, INSERM, Université de Nantes, Université d’Angers, Angers, France; ^3^ Laboratoire de Cytogénétique et Biologie Cellulaire, CHU Rennes, Rennes, France; ^4^ SERGOH, CHU Angers, Angers, France; ^5^ Département de Neurochirurgie, CHU Angers, Angers, France

**Keywords:** glioblastoma, whole genome duplication, aneuploidy, chromothripsis, SNP arrays

## Abstract

Glioblastoma, the most frequent and lethal form of glioma, displays chromosome instability and recurrent somatic copy number alterations (SCNA). Chromothripsis and whole genome duplication (WGD) have been recently identified in cancer. In the present study, we analyzed SCNA and determine the ploidy pattern in 123 *IDH*-wild-type glioblastomas, using SNP array data. WGD and chromothripsis events were validated using, respectively, FISH and CTLPScanner. WGD was detected in 11.4% glioblastomas (14/123) and was associated with *TP53* mutation (*p* = 0.0068). It was an early event occurring after the recurrent SCNA observed in diffuse high-grade gliomas. Glioblastomas with WGD were more aneuploid compared to glioblastomas without WGD (*p* < 0.0001). Chromothripsis occurred in 29.3% glioblastomas (36/123) and mostly affected chromosomes 7, 9 and 12, with amplification of oncogenes (EGFR, *MDM2*/*CDK4*), and homozygous deletion of tumor suppressor genes (*CDKN2A*). There was a significant association between chromothripsis and gene rearrangement at a given locus. WGD is an early genetic event significantly associated to *TP53* mutation and leading to chromosome instability and aneuploidy in *IDH*-wild-type glioblastoma. Chromothripsis recurrently targets oncogenes and tumor suppressor genes that are key players in gliomagenesis and tumor progression. The occurrence of chromothripsis points to underlying gene rearrangements (including gene fusions), potential therapeutic targets in glioblastoma.

## INTRODUCTION

Glioblastoma (GBM) is the most frequent and most aggressive form of diffuse glioma. The prognosis is very poor, with a median overall survival of 15 months after maximum safe resection and radiochemotherapy [1]. GBM is one of the most genetically unstable cancers. It is characterized by numerous chromosome (chr) copy number alterations (CNA), such as chr 7 gain, chr 9p loss, and chr 10 loss, along with *CDKN2A* homozygous deletion (chr 9p21) and *EGFR* amplification (chr 7p11) [2]. Chromosome instability (CIN) may be the cause or the consequence of GBM development. In high-grade diffuse gliomas, CIN may initiate tumorigenesis.

Even if somatic CNA (SCNA) have been well described in GBM, general mechanisms leading to massive rearrangements implicating several, if not all, chrs are not well understood. Among these mechanisms, whole genome duplication (WGD) leads to tetraploidization of cells, which may pave the way to aneuploidy [3]. Several studies have shown that WGD is an early event in tumorigenesis. Tetraploid cells have been observed in the transition from premalignant to malignant disease (e.g., Barrett’s oesophagus, cervical intra-epithelial neoplasia), suggesting that a genome-doubling event can be a driver of tumorigenesis [4–6]. The frequency of WGD varies across tumor types; WGD is more frequent in carcinomas, with an incidence >50% in colorectal, breast, lung, ovarian and oesophageal carcinomas, but has been reported in 25% GBM [7]. In a recent pan-cancer analysis, tumors with WGD exhibited a greater number of SCNA, with marked chr losses occurring after WGD [8]. Carter *et al.* showed that WGD increased tolerance to subsequent CIN events [7]. Furthermore, the authors observed that genome-doubled samples were associated with significantly older age at diagnosis and greater incidence of tumor recurrence. No study has correlated WGD with age and overall survival in GBM.

CT is a recently described phenomenon whereby massive chr shattering occurs during a single (mitotic) event [9]. It is a cataclysmic event in which a single or a few chrs are shattered and then randomly reassembled in a derivative chr, sometimes with additional circular double-minute chrs. The latter may include an oncogene whose amplification may confer a growth advantage to the cell. Conversely, some chr pieces may be lost. In addition, chr rearrangements can juxtapose the coding sequences of two genes, leading to novel fusion genes with oncogenic potential. 2–3% of all cancers show evidence of massive remodelling of a single or a few chrs, with tens to hundreds genomic rearrangements [9]. CT has recently been proposed as a novel mechanism for genetic instability and cancer development. It has been reported in a broad range of tumors (prostate carcinomas, multiple myelomas, GBM, medulloblastomas, neuroblastomas) [10, 11]. The mechanisms underlying CT are not yet known. To date, only two studies, both using the TCGA-GBM cohort, have analyzed CT occurrence in GBM [8, 12]. Zack *et al.* showed that CT events mostly involved chrs 9 and 12, and were associated, respectively, with deletion of *CDKN2A* (9p21) and co-amplification of *MDM2*/*CDK4* (12q15/12q14) [8]. A recent study by Furgason *et al.* on 12 GBM using whole genome sequencing established a link between CT and amplification of known oncogenes (e.g., *EGFR*, *MDM4*, *MDM2/CDK4*) [12].

The occurrence and impact of WGD and CT have never been studied in a large series of primary GBM independent from the TCGA cohort. Herein, we investigated WGD and CT events in a series of 123 primary *IDH*-wild-type GBM and correlated the results with the survival data.

## RESULTS

### Patient characteristics

Median age at diagnosis was 63 years old (range: 22 to 84 years). Male-to-female ratio was 1.56. Median OS was 14.2 months and median PFS was 7.7 months.

### Immunohistochemistry

We performed IHC against IDH1-R132H mutant protein on the whole cohort. No GBM expressed IDH1-R132H mutant protein arguing for the diagnosis of primary *IDH*-wild-type GBM.

### Sanger sequencing

No *IDH1*/*2* mutation was detected supporting the diagnosis of *IDH*-wild-type GBM according to the 2016 WHO classification [2]. Thirty-four cases out of 123 (27.6%) harbored *TP53* mutation.

### SNP array analysis

**Figure 1:**
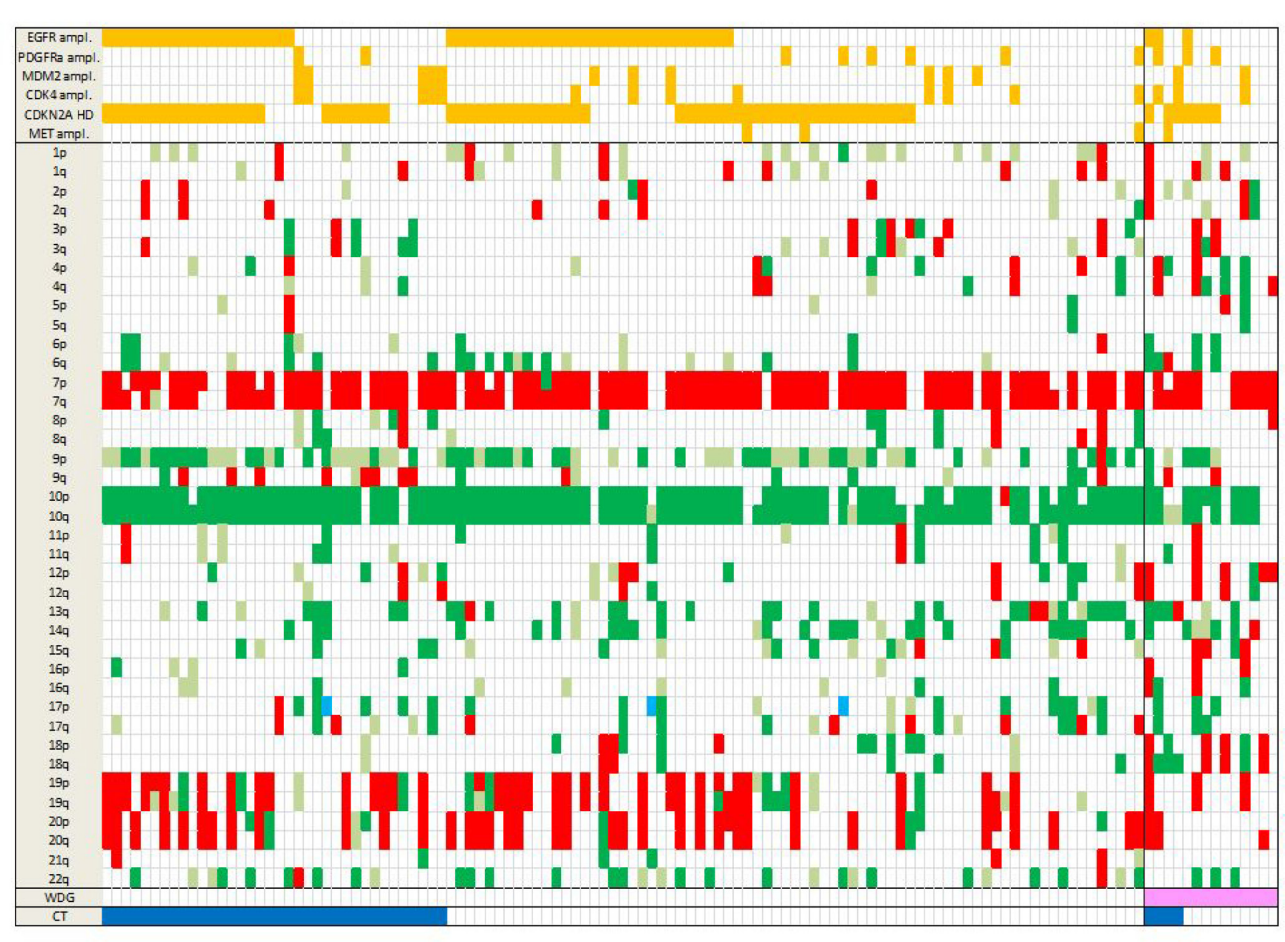
Landscape of SCNA in the 123 GBM Total chr losses are in dark green, partial losses in light green, chr gains in red and copy neutral LOH in light blue. Amplifications and homozygous deletions of key genes in GBM are shown in orange (top rows). WGD is in pink and CT in dark blue (bottom rows). Note that WGD cases present marked aneuploidy with many chr losses. Abbreviations: SCNA, somatic copy number alterations; chr, chromosome; ampl, amplification; HD, homozygous deletion; LOH, loss of heterozygosity; WGD, whole genome duplication; CT, chromothripsis.

SNP array was performed on the 123 cases. The most frequent CNA were chr 10q loss (106/123, 86.2%), chr 7p gain (92/123 (74.8%), including 89 cases with additional 7q gain), chr 9p loss (77/123, 62.6%), and chr 19 and 20 co-gain (23/123, 18.7%) (Figure 1). *CDKN2A* homozygous deletion and *EGFR* amplification were the most frequent recurrent focal alterations, detected in 71 (71/123, 57.7%) and 53 cases (53/123, 42.3%), respectively. *CDK4* amplification was found in 19 GBM (16/123, 15.4%), *MDM2* amplification in 11 tumors (11/123, 8.9%), and *MET* amplification in 4 GBM (4/123, 3.2%).

### WGD is present in 11.4% of GBM

In most cancers, WGD corresponds to tetraploidy, which is a type of polyploidy in which a single cell has two sets of chromosomes (4n or 92 chromosomes) instead of one (2n or 46 chromosomes).

SNP array data analysis showed that most GBM (109/123, 88.6%) had a near-diploid karyotype with a number of chrs between 39 and 52, and an average estimated ploidy of 2.03. However, some GBM (14/123, 11.4%) presented a near-tetraploid DNA content, with a number of chrs oscillating between 58 and 92, and an average estimated ploidy of 3.46, representing WGD (Supplementary Figure 1).

In comparison with near-diploid GBM, WGD GBM displayed more chr losses (but no gain) and allelic imbalances. In near-diploid GBM, a ploidy status near 2 was observed in 76.7% cases whereas a ploidy status near 4 was observed in only 22.4% WGD GBM (*p <* 0.0001). There was a tendency for chr losses after genome doubling. This observation is supported by the average estimated ploidy of 3.46 in near-tetraploid GBM. Hence, GBM with WGD presented with a larger overall number of SCNA.

**Figure 2:**
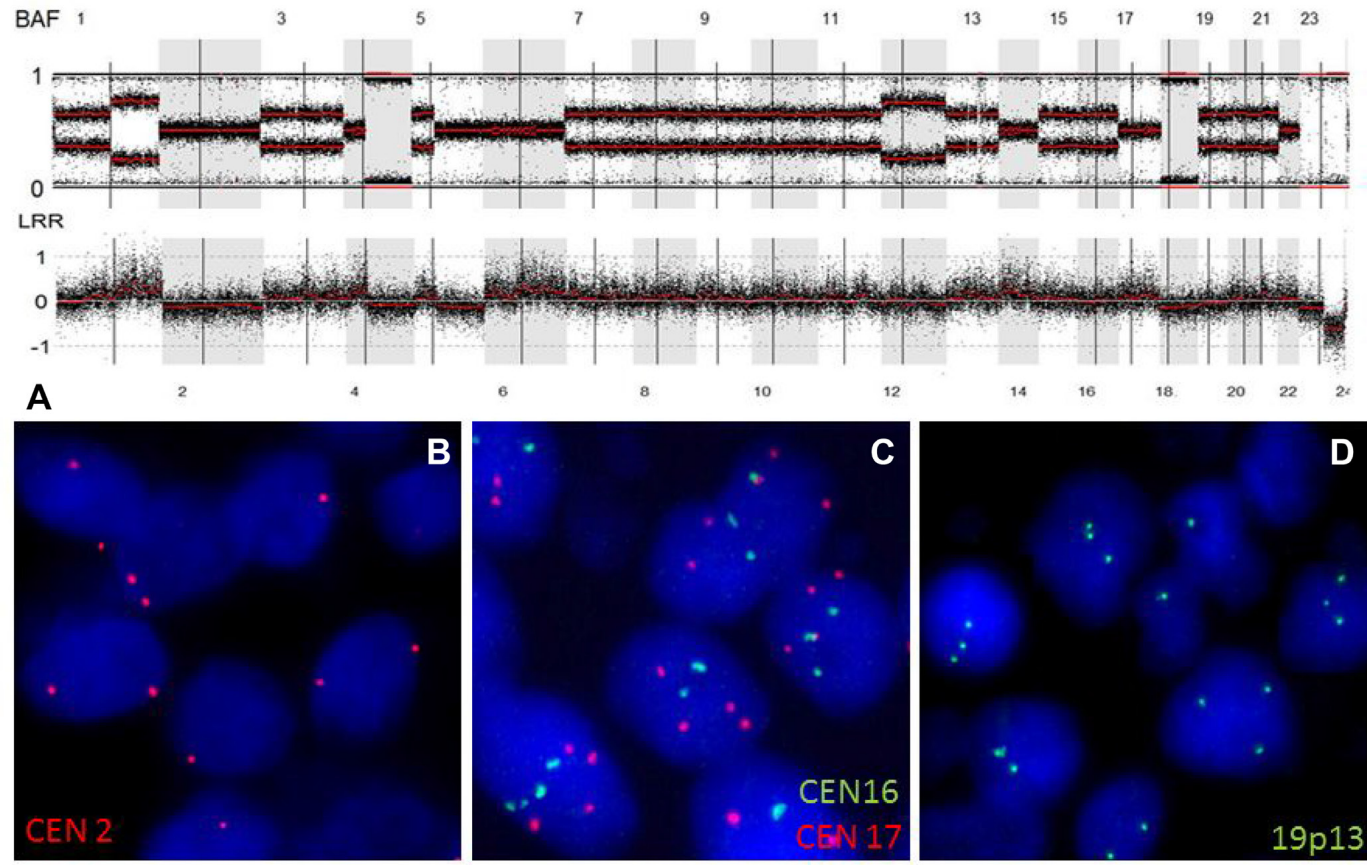
WGD in an *IDH*-wild-type GBM (**A**) SNP array profile (GAP method) showing a near tetraploid genome characterized by marked allelic imbalance. Chr 2 displayed a LogR ratio below 0 (2 DNA copies), with a heterozygous allelotype; chr 17 had a LogR ratio above 0 (4 DNA copies), with a heterozygous allelotype; chrs 16 and 19 displayed a LogR ratio near 0 (3 DNA copies). (**B**) FISH picture showing the presence of two copies of chr 2 (red signals). (**C**) FISH picture showing the presence of four copies of chr 17 (red signals) and three copies of chr 16 (green signals). (**D**) FISH picture showing the presence of three copies of chr 19q13 (green signals). Abbreviations: BAF, B-allele frequency; LRR, LogR Ratio; CEN, centromere.

We used a FISH approach to confirm WGD in 6 GBM (out of 14) with a near-tetraploid karyotype (Supplementary Table 2). At least three chrs per sample were tested. For all the chrs tested, the copy number was in accordance with that observed on GAP analysis. For case GBM49-003, the SNP array data showed that there were two copies of chr 2 with a heterozygous allelotype, there were three copies of chr 19 with an allelic imbalance, and there were four copies of chr 17 with a heterozygous allelotype. The same copy numbers were detected using FISH (Figure 2).

**Figure 3:**
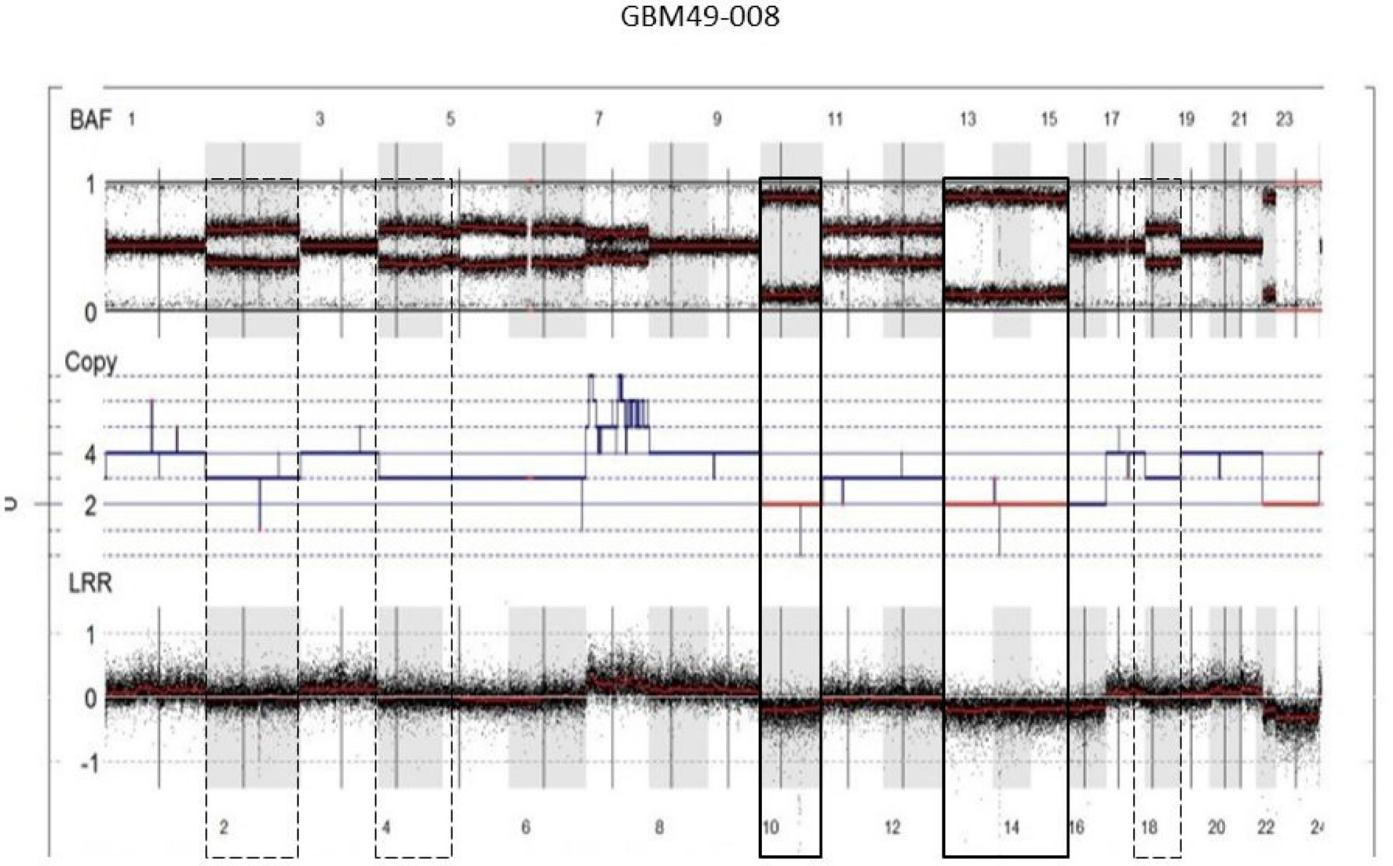
Chronology of genetic events in WGD GBM SNP array profile (GAP method) of a near-tetraploid GBM showing two types of chr losses: chr losses with a homozygous allelotype (2 DNA copies instead of 4; e.g., chrs 10, 13q, 14q) (solid boxes) and chr losses with a heterozygous allelotype (three DNA copies; e.g., chrs 2 and 4) (dashed boxes). According to these observations, chrs 10, 13q and 14q were lost before WGD occurrence, and chrs 2 and 4 were lost after genome doubling. Abbreviations: BAF, B-allele frequency; LRR, LogR Ratio; CEN, centromere.

Moreover, analysis of the SNP array profiles helped retrace the chronology of the genetic events in GBM. For example, a near-tetraploid GBM presented two types of chr losses: chr losses with a homozygous allelotype (2 DNA copies instead of 4) (e.g., chrs 10, 13q and 14q) and chr losses with a heterozygous allelotype (e.g., chrs 2 and 4) (Figure 3). According to these observations, chrs 10, 13q and 14q were lost before WGD occurrence, and chrs 2 and 4 were lost after genome doubling. In our cohort, 73.3% chr losses (11/15 cases) did not present loss of heterozygosity (LOH), hence occurred after WGD. Inversely, chr 10 loss and chr 9p loss occurred before WGD (8/8 cases and 6/6 cases, respectively) in our cohort. This observation suggests that WGD is an early event and most likely contributes to CIN. For 3 WGD GBM (out of 14), the recurrent tumor was analyzed by SNP array and all 3 cases displayed stable WGD profiles, without significant additional losses or gains (Supplementary Figure 2).

We did not find any association between WGD and common SCNA in GBM (Supplementary Table 3). The frequency of specific arm-level SCNA (e.g., 7p gain, 9p loss) was not significantly different in diploid vs tetraploid GBM, except for chr 10q loss, which was less frequent in WGD GBM (*p =* 0.0044).

In our cohort, even if the difference was not statistically significant, WGD GBM tended to occur at a younger age compared to near-diploid GBM (median age 55 *vs* 63 years, respectively). There was no association between WGD and survival times (OS and PFS; OS: 13.3 months in WGD cases *vs* 14.2 months in diploid cases).

### 29.3% of GBM harbored CT patterns

**Figure 4:**
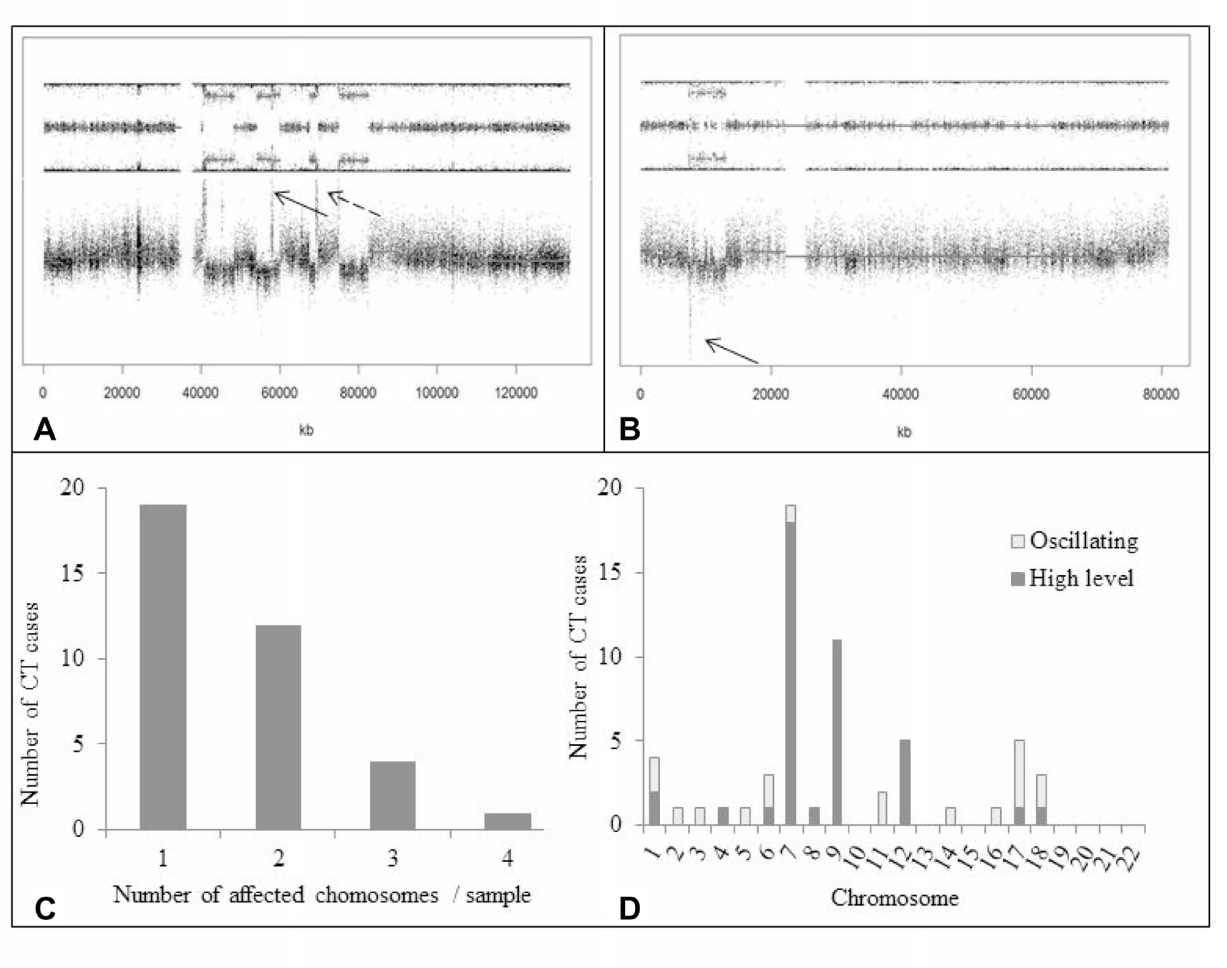
Occurrence of CT (**A**) SNP array profile (GAP method) showing a CT pattern on chr 12q with multiple amplifications involving, among others, *MDM2* and *CDK4* genes (dotted and solid arrows, respectively). Note interspersed loss and retention of heterozygosity with no more than three different copy number states except for focal amplifications. (**B**) SNP array profile (GAP method) showing a CT pattern on chr 17p with *TP53* homozygous deletion (arrow). (**C**) Number of chrs affected by CT per GBM sample. In most cases, CT involved only one or two chrs. (**D**) CT occurrence across the whole genome in the 123 GBM. High-level event indicates gene amplification (³ 4 copies) or homozygous deletion (no copy); oscillating pattern indicates alternating copy number states (1, 2 or 3) without amplification or homozygous deletion of genomic regions. High-level events were more frequent than oscillating patterns. CT mostly involved chrs 7, 9, and 12, which harbor key oncogenes or tumor supressor genes in GBM. Abbreviations: CT, chromothripsis.

**Figure 5:**
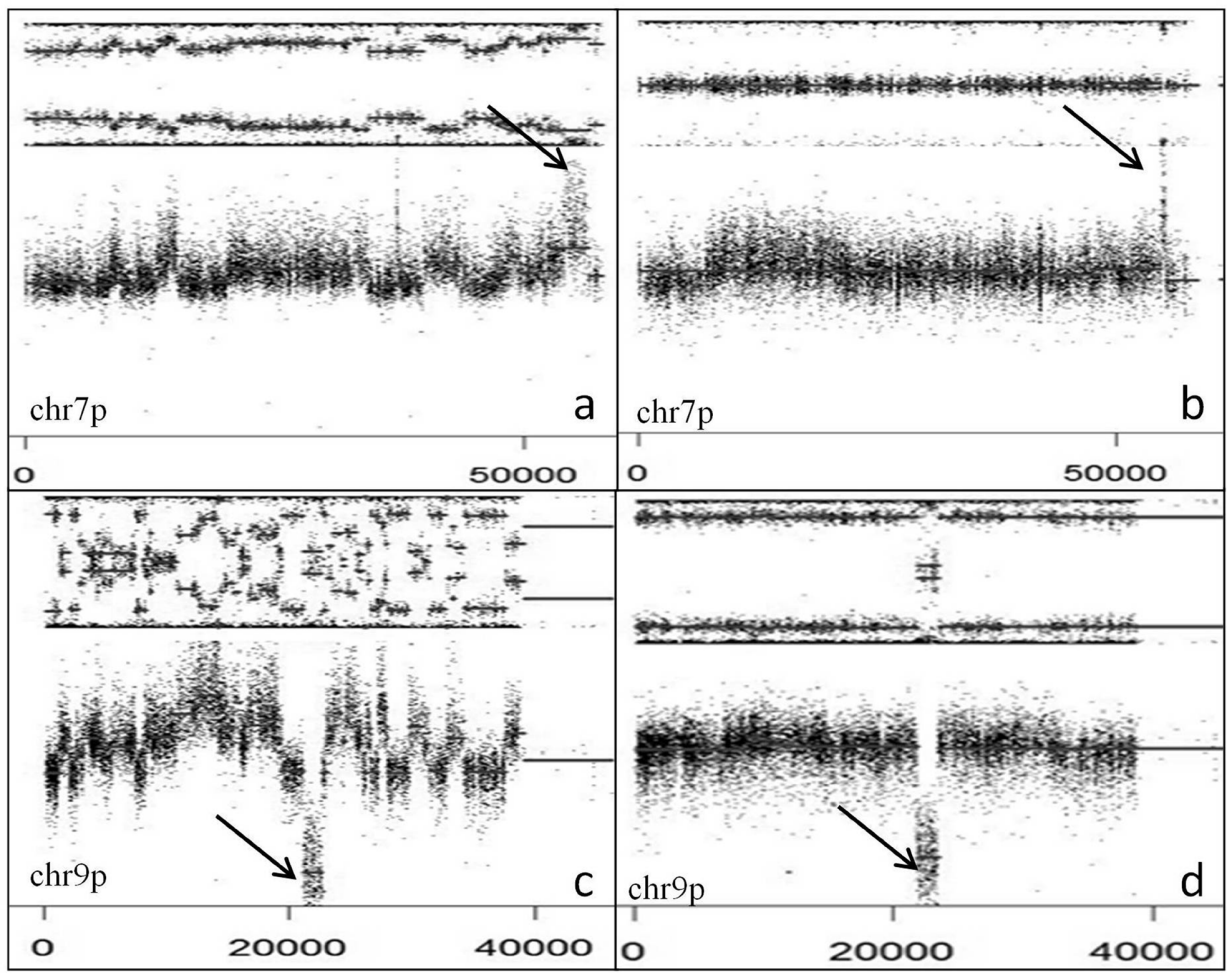
*EGFR* amplification and *CDKN2A* deletion with or without CT SNP array profiles (GAP method) showing EGFR amplification (arrow) and chr 7 gain with (**A**) or without (**B**) CT. *CDKN2A* homozygous deletion (arrow) and chr 9p loss with (**C**) or without (**D**) CT. Note interspersed loss and retention of heterozygosity with no more than three different copy number states except for focal amplification or homozygous deletion.

Out of 123 GBM, 36 (29.3%) presented a CT pattern (Figures 1 and 4). CT events were defined as 1) the presence of at least 10 genomic rearrangements per chr arm with such rearrangements occurring in no more than 4 chrs in a given tumor, 2) a clustering of breakpoints, and 3) interspersed loss and retention of heterozygosity. Thirty-one of the 36 cases (86.1%) presented a CT pattern only on one or two chrs whereas 5 cases presented a CT pattern on three or four chrs (Figure 4). CT events mostly affected genomic regions harboring common driver alterations in GBM (Table 1). Indeed, CT was detected on chrs 7, 9 and 12, involving *EGFR* (7p11), *CDKN2A* (9p21), and *MDM2*/*CDK4* (12q15/12q14) loci, respectively. Seventy-five percent GBM (27/36) displayed CT at least at one of these loci. The presence of a CT pattern in a given genomic region was highly associated with detection of gene amplification or homozygous deletion in that region. CT patterns were found at the *EGFR* locus in 15 patients (41.6%) and at the *CDKN2A* locus in 14 cases (38.8%). In our series, 15 of 52 (28.8%) GBM harboring *EGFR* amplification presented a CT pattern affecting the *EGFR* locus (*p <* 0.0001) and 14/72 (19.4%) GBM with *CDKN2A* homozygous deletion displayed a CT pattern on chr 9p21 (*p =* 0.0003) (Figure 5). CT also involved the *TP53* locus in two cases harboring *TP53* homozygous deletion. We also observed CT patterns at other loci displaying amplification of *MDM4* (1q32), *MET* (7q31) or *PDGFRa* gene (4q12) (one case each). Finally, 6 GBM (out of 123, 4.8%) had *CDK4* amplification (12q14.1) and 13 GBM (13/123, 10.5%) presented co-amplification of *MDM2* and *CDK4* genes. More than half of those co-amplified cases (7/13; 53.8%) presented a CT pattern at the *MDM2* and *CDK4* loci (*p <* 0.0001) (Figure 3). In 5 of these 7 cases, we observed additional amplifications with a CT pattern on chr 12 (Supplementary Table 4). Some amplicons implicated oncogenes such as *KRAS* (l2p12) and *HMGA2* (12q14). We also noted amplification of *STAT2* gene (12q13.3) in one case.

**Table 1:** Genomic regions displaying CT patterns

Chromosome	Start region	End region	Size (Mb)	Number of cases	Candidate genes
**7p11**	**52020592**	**55481389**	**3.5**	**15**	*EGFR*
**9p21**	**21255150**	**24346050**	**3.1**	**14**	*CDKN2A*
**12q14-q15**	**58131014**	**69506488**	**11.4**	**5**	*MDM2, CDK4*
**6q25.3-q26**	**159550853**	**162731343**	**3.2**	**3**	*PARK2*
**1p34.2-p32.3**	**43602321**	**52255483**	**8.7**	**2**	*CDKN2C*
5q31.3-q35.2	140225908	174555605	34.3	2	
11q12.3-q13.4	62061349	73477045	11.4	2	*HRASLS5*
17p13.1-p12	7543994	13031942	5.5	2	*TP53*
17q21.33-q24.3	49168871	68746950	19.6	2	
1p36.11-p33	24418768	47891361	23.5	1	
1q32.1-q32.3	202967491	213629078	10.7	1	*MDM4*
2q23.1-q24.1	149198831	159778783	10.6	1	
3q27.3-q29	186158483	197838262	11.7	1	
4q11-q22.3	52682617	95817017	43.1	1	*PDGFRA*
8q24.13-q24.22	124566356	131895923	7.3	1	*PVT1*
14q11.2-q32.33	20452460	107287663	86.8	1	
16q23.1-q23.3	75659849	82854894	7.2	1	
17q11.1-q21.31	25295032	42989088	17.7	1	*NF1*
18q12.2-q23	33205897	77896242	44.7	1	
18q21.1-q21.31	47996600	54815307	6.8	1	*TCF4*
18q21.33-q22.1	61394140	66558392	5.2	1	
7q11.23-q31.2	72283565	116438418	44.2	1	*MET*

Table summarizing genomic regions affected by CT in the 123 GBM. These regions are classified from most to least frequently involved.

CT also underlies oscillating rearrangements without homozygous deletion or gene amplification (18/59 CT regions) as observed for chrs 6 (3 cases), 11 (2 cases), and 17 (3 cases; Supplementary Figure 3). Of note, in our cohort some chrs were never affected by CT (chrs 19 to 22).

We performed *TP53* gene sequencing on the whole cohort. Thirty-four cases out of 123 (27.6%) harbored *TP53* mutation. Among the 36 GBM with CT, 6 (16.7%) displayed *TP53* mutation. There was no association between CT occurrence and *TP53* mutation (*p =* 0.13). However, 9 tumors out of the 14 WGD GBM (64.3%) harbored *TP53* mutation. The association between WGD and *TP53* mutation was statistically significant (*p =* 0.0068).

## DISCUSSION

The study presented herein thoroughly describes two distinct genetic events that may play a key role in the initiation and/or progression of GBM. We studied WGD and CT in a large series of 123 primary *IDH*-wild-type GBM independent from the TCGA GBM cohort. WGD is a common event in human cancer, with an average frequency of 37%. The frequency is highly variable across tumor types; carcinomas from the ovary, breast, lung, and esophagus have an incidence of genome doubling of about 50% whereas WGD has not been observed in leukemia [7]. Two studies reported a frequency of WGD in TCGA GBM of 25% and 11%, respectively [7, 8]. Our frequency of 11.4% is consistent with these previous findings. Copy number losses occurring in a diploid cell will result in LOH. This LOH will leave a permanent footprint in the genome, persisting after a genome-doubling event. In contrast, losses occurring after genome doubling are less likely to exhibit LOH [13]. In our cohort, the small proportion of LOH observed on the SNP array profiles suggests that WGD was an early genetic event, potentially leading to CIN. However, some events such as chr 10 loss and chr 9p loss appeared before genome doubling. This observation suggests that key SCNA in GBM appear before genome doubling; WGD may generate CIN, with occurrence of many subsequent chr losses. This hypothesis is consistent with the results of Carter *et al.*, who showed that most common SCNA in GBM occur before WGD [7]. In Zack *et al.*’s work, WGD was inferred to occur earliest relative to focal SCNA among lineages where WGD was common (ovarian, bladder and colo-rectal cancers) and after most focal SCNA in lineages in which WGD was least common (GBM, renal cell carcinoma) [8].

We showed that GBM with WGD were more aneuploid compared to GBM without WGD (*p <* 0.0001). Dewhurst *et al.* showed that tolerance of aneuploidy was enhanced in tetraploid clones from a colon cancer cell line but was a rare event in diploid cells [13]. Daughter cells derived from a parental cell that had undergone a segregation error frequently died or underwent cell-cycle arrest. Daughter cells arising from tetraploid clones after a segregation error died or arrested less frequently, with the majority continuing through mitosis in the subsequent cell cycle [13]. WGD is a lot more common in plants compared to animals. Polyploidy is usually an evolutionary dead end, except in abnormal circumstances when polyploids might have an evolutionary edge over non-polyploids [3]. Robust growth of tetraploid cells may be via evolution toward a more favorable karyotype [14]. After genome doubling, massive chr losses occur (leading to intermediate ploidy level), which may allow tumor cells to reach a genomic equilibrium that favors survival and growth. Dewhurst *et al.* demonstrated that genome doubling is an early event in some colorectal cancers, permitting the evolution of more genomically complex, subtetraploid, higher-stage tumors [13]. Polyploidy confers adaptive potential to the (tumor) cells especially during periods of extreme stress by providing mutational robustness. Polyploidy-related changes in gene expression and epigenetics may facilitate or accelerate adaptation (to a new or changing environment) [3]. WGD may contribute to intra-tumor heterogeneity, a substrate for selection and tumor evolution [15]. Tumor cells with WGD may become resistant to radiation therapy and chemotherapy and hence, give rise to tumor recurrence. Of note, the recurrent tumors we analyzed still displayed WGD suggesting that genome doubling indeed confers a selective growth advantage to the cells.

Different mechanisms may lead to the emergence of tetraploid cells in tumors, such as cellular stress, abnormal cell division, or telomere shortening [16]. Mutations affecting the DNA damage response (e.g., *TP53* mutation) enable polyploid cells to further proliferate. The propagation of tetraploid cells is inhibited by p53, which arrests the cell cycle in G1 phase [17]. However, p53 inactivation, such as present in 90% of GBM, and gene overdosage (in tetraploid cells) confer tolerance for SCNA [13], allowing tetraploid cells to survive. This is in accordance with our results showing a statistically significant association between *TP53* mutation and WGD occurrence (*p =* 0.0068).

In our cohort, there was no association between WGD and CT. Only 4 out of 14 WGD GBM displayed CT (28.5%). The size of the cohort may be too small to show such an association. Mardin *et al.* showed in a model cell line that hyperploidization preceded CT, implicating hyperploidization as a “risk factor” for CT *in vivo* [18]. They showed that the tetraploid cell lines exhibited a significantly higher level of CNA as those derived from the diploid cell line. They observed highly clustered CNA in nine cases, all of which arose in hyperploid lineages. Their analysis indicated an increased rate of CT events in hyperploids compared to diploids (*p* < 0.05). Mardin *et al.* showed in human Sonic-hedgehog pathway-driven medulloblastoma specimens that CT indeed occurred significantly more often in hyperploid compared to diploid tumors [18]. The higher tolerance for CIN in tetraploids might explain why CT could be associated with hyperploid cells but not with diploid cells. Polyploidy buffers the effects of partially recessive deleterious mutations. Transient tetraploidy can, by generating and buffering aneuploidy, result in long-term adaptation. According to Dewhurst *et al.*, a genome-doubling event could represent a macro-evolutionary leap in tumors, which precipitates and sustains extensive chromosomal rearrangements such as CT [13]. Large phenotypic leaps facilitate rapid evolution to novel selection pressures [19]. CT potentially constitutes a mechanism by which aggressive, spontaneous tumor, such as GBM, could arise in a relatively short period of time [12]. Of interest, we studied 43 cases of *IDH*-mutated 1p/19q-codeleted oligodendrogliomas using SNP arrays and none displayed WGD or CT (data not shown). The better prognosis of such tumors might be related to a more stable genome and the absence of WGD or CT event.

In our cohort, WGD was associated with a younger age at diagnosis (not statistically significant) but had no impact on survival. In Dewhurst *et al.*’s study, WGD was independently predictive of poor relapse-free survival in early-stage colo-rectal cancer [13]. Analyses in larger GBM series will be needed to determine the association between WGD and age or prognosis.

The second genetic event studied herein is CT. In our cohort, CT was detected in 29.3% of cases, consistent with the findings of the TCGA studies [9, 12, 20]. In accordance with previous works [12], CT mostly involved chrs 7, 9 and 12, with associated *EGFR* amplification, *CDKN2A* homozygous deletion and *CDK4*/*MDM2* co-amplification, respectively. Our results show that CT underlies amplification of oncogenes and homozygous deletion of tumor suppressor genes. In our cohort, 28.8% of *EGFR*-amplified GBM presented a CT pattern at the *EGFR* locus (7p11.2) (*p <* 0.0001), and 19.4% of *CDKN2A*-deleted GBM harbored a CT pattern at the *CDKN2A* locus (9p21.3) (*p =* 0.0003). Yang *et al.* studied a cohort of 16 TCGA GBM using whole genome sequencing [21]. Four of 12 patients (33%) harboring *CDKN2A/B* deletion displayed complex rearrangements at the 9p21 locus and 8/14 GBM (57%) with *EGFR* amplification presented complex rearrangements at 7p11. Taken together, these data suggest that complex rearrangements including CT are responsible for *EGFR* amplification and *CDKN2A/B* deletion.

Many studies suggest that CT is a phenomenon leading to chr rearrangements (including gene fusions) implicating yet to discover driver genes. Few fusion genes have been described in high-grade gliomas. Singh *et al.* reported *FGFR3*-*TACC3* and *FGFR1*-*TACC1* gene fusions in 3% GBM [28] The *FGFR3*-*TACC3* fusion might be suspected on SNP arrays by detecting focal gains involving *FGFR3* (chr 4p16.3). The identification of such fusion genes allowed the development of targeted therapies, with promising preliminary results [22]. Frattini *et al.* described recurrent in-frame fusions involving *EGFR* (chr 7p11) and different partners (*SEPT14, PSPH) in 7% GBM* [30]. Of note, in our cohort, one case harboring a CT pattern on chr 7p11 and *EGFR* amplification displayed an *EGFR*-*SEPT14* fusion. Four additional cases displayed gene fusions recurrently implicating *EGFR*, *SEPT14* or *VOPP1* genes; those fusions, detected by RNA sequencing, were validated by RT-PCR and Sanger sequencing (data not shown, manuscript in preparation). Recently, a fusion between *NAB2* and *STAT6* genes (chr 12q13.3) associated with a focal 12q13 amplification has been reported in a 29-year-old patient with an *IDH1*-mutant GBM [23]. Chr 12 often harbors numerous genomic rearrangements, which are associated with the occurrence of gene fusions [24, 25]. In our cohort, out of 13 GBM with *MDM2/CDK4* co-amplification, 7 had additional focal amplifications on chr 12q. In view of those results, focal gains or amplifications may indicate the presence of yet-to-identify gene fusions. Of particular interest, Parker *et al.* described a fusion between *RELA* and *C11orf95* resulting from CT on chr 11q13 in supratentorial ependymomas with a dismal prognosis [26]. CT has also been associated with gene fusions in other tumor types (medulloblastoma, small cell lung carcinoma, diffuse large B cell lymphoma) [27–29]. The presence of gene fusions in CT regions remains to be thoroughly investigated as it may open new therapeutic avenues in GBM.

Numerous mechanisms for CT occurrence have been proposed, including telomere attrition (leading to breakage-fusion-bridge cycles), aberrant mitosis producing micronuclei, and premature chr compaction [12, 30]. Chr segregation errors during mitotic cell division can entrap DNA within a micronucleus. Micronucleated chrs are susceptible to shattering during the next mitosis generating multiple distinct DNA fragments [30]. In the absence of functional p53, DNA double-strand break repair ensues through error-prone non-homologous end joining, which directly links multiple fragments together in a haphazard manner by ligation [30]. Interestingly, tetraploid cells display frequent chr mis-segregation, supporting the hypothesis that WGD is a risk factor for CT.

With regard to the limitations of the study, we only tested 4 chromosomes per sample by FISH to confirm WGD. Isolation of tumor cells from fresh tissue would have allowed analysis of whole nuclear DNA content by flow cytometry. However, FISH technique based on the use of centromere probes represents a sensitive method for detecting chromosome aneuploidies [31]. Regarding CT, more stringent criteria can be used to identity CT patterns based on whole-genome paired-end DNA sequencing data [32]. Nonetheless, SNP arrays allow for the detection of the major features of CT: clustering of breakpoints, regularity of oscillating copy-number states, and prevalence of regions with interspersed loss and retention of heterozygosity [32]. A consensus on which criteria are to be met to define CT has yet to be reached.

In conclusion, we demonstrated that WGD was an early event associated with *TP53* mutation and leading to aneuploidy in *IDH*-wild-type GBM. Those are novel findings in GBM pathogenesis. Our results concur with those of others that the occurrence of CT points to underlying gene rearrangements, potential key drivers in gliomagenesis. According to Notta *et al.*, SCNA from CT are essentially clonal, suggesting that these events are also sustained early in tumorigenesis [33]. Cancer genome instability drives tumorigenesis and tumor growth. It also fuels intercellular heterogeneity and tumor evolution. Genomic instability may drive branched evolution of tumor cells contributing to drug resistance. According to the Big Bang model proposed by Sottoriva *et al.* in colorectal cancer, there are uniformly high levels of intra-tumoral heterogeneity throughout the neoplasm [34]. Both public (clonal) and most private (subclonal) alterations occur early in tumor growth [34]. The Big Bang model implies punctuated clonal evolution (*vs* sequential clonal evolution) such as mutational bursts or CT. Such a model might explain GBM intra-tumoral heterogeneity seen at diagnosis. Even though the initial event in neoplastic transformation is still unknown, assessing spatial heterogeneity via multiple sampling and genomic profiling of distinct tumor regions within a GBM may help designing effective multidrug therapeutic strategies.

## MATERIALS AND METHODS

### Patients and tumor samples

One hundred and twenty-three patients were selected according to the following criteria: 1) histopathological diagnosis of primary GBM, *IDH*-wild-type according to the 2016 WHO classification [2], 2) available fresh-frozen tissue, 3) available clinical and survival data, and 4) written informed consent, with approval of the research ethics committee of Angers University Hospital. Fresh-frozen tumor samples, with at least 60% of tumor cells, were obtained from Angers Hospital biobank (CRB, Center for Biological Resources).

### Immunohistochemistry

Immunohistochemistry (IHC) against IDH1-R132H mutant protein (clone H09, 1/200, Dianova) was performed on the 123 cases. Four μm-thick formalin-fixed paraffin-embedded (FFPE) tissue sections were treated with the Bond polymer Refin Detection DS98000 kit (Leica Biosystem) and immunostained in the Leica Bond-III^®^ (Leica Biosystem). IDH1-R132H mutant protein expression was scored as positive when ≥10% immunopositive tumor cells were present.

### Sanger sequencing

Tumor DNA was extracted from fresh-frozen tissue using the NucleoSpin^®^ Tissue kit (Macherey-Nagel) according to the manufacturer’s instructions. Purified DNA was quantified using the Nanodrop ND-1000^®^ (NanoDrop Technologies). The targeted genes, *IDH1*/*2* and *TP53*, were analyzed using Sanger sequencing. The primers are listed in Supplementary Table 1. Cycle parameters comprised 95° C × 10 min; 40 cycles of 94° C × 30 s, 60° C × 45 s, 72° C × 45 s; 72° C × 10 min. PCR products were purified using the PCR NucleoFast^®^ plate (Macherey-Nagel). Purified PCR products were sequenced using the Big-Dye^®^ Terminator Cycle Sequencing Ready Reaction (Applied Biosystems). Sequences were purified with Sephadex Superfine G50 (Sigma Aldrich) and analyzed on an ABI Prism 3710 DNA Analyzer (Applied Biosystems).

### SNP array and copy number analysis

SNP array was performed on the 123 samples. Tumor DNA was extracted using the Nucleospin Tissue Kit (Macherey Nagel) and quantified using Qubit dsDNA BR Assay Kit (Life Technologies). Tumor DNA was hybridized with Infinium CytoSNP-850 K Illumina Beadchips (Illumina**)** according to the manufacturer’s instructions. SNP arrays were scanned on an iScan (Illumina) and data were processed using the genotyping module in Genome Studio v2011.1 (Illumina) to calculate B-allele frequencies (BAF) and logR ratios. GAP method was used to call SCNA and to determine the number of chrs and the ploidy pattern (near-diploid, near-tetraploid) for each tumor [35]. The GAP method relies on two parameters to estimate tumor ploidy: 1) the DNA index for a tumor genome estimated as an average copy number in a genome divided by 2, and 2) the chromosome counts in a tumor genome estimated as a sum of copy numbers at pericentric regions of each chromosome arm. The calling of SCNA was based on the analysis of logR and allelic ratios [36]. The purity of the samples was assessed using BAF values. SNP array data are deposited in public repository ArrayExpress under the accession number E-MTAB-6371.

### CT identification

CT events were detected using segmented data (LogR ratio, BAF value) from the SNP arrays. They were identified following the three criteria described by Korbel and Campbell [32]:

1) There were at least 10 genomic rearrangements per chr arm with such rearrangements occurring in no more than 4 chrs in a given sample,
2) There was a clustering of breakpoints,
3) There was interspersed loss and retention of heterozygosity with no more than two to three different copy number states (except for focal amplification or homozygous deletion (involving key cancer genes)).

After manual screening according to the above-mentioned criteria, a validation analysis was performed on segmented data using CTLPScanner, a web server for detection of CT patterns [37]. Scanner parameters and thresholds were as follows: genome assembly = hg19; copy number status change times ≥ 10; log10 of likelihood ratio ≥ 6; minimum segment size = 5 Kb.

### WGD validation by FISH

FISH testing was performed on 4-µm sections of FFPE tumor samples. We used Vysis CEP2 (D2Z1) and CEP17 (D17Z1) SpectrumOrange probes (targeting the centromere of chr 2 and chr 17, respectively) and Vysis CEP16 (D16Z3) and LSI 19p13 SpectrumGreen probes (targeting the centromere of chr 16 and the chr 19p13 locus, respectively) according to the manufacturer’s instructions (Abbott). Two cytogeneticists (FD, MABR) analyzed the slides using a fluorescence microscope (Axioskop2, Axio Imager Z2, Zeiss) and Isis imaging software (Metasystems). At least 100 non-overlapping tumor nuclei were examined per case.

### Statistical analyses

The khi^2^ test was used to compare the distribution of categorical variables and unpaired *t*-test or Mann–Whitney test was used to compare continuous variables. Overall survival (OS) was defined as the time between histopathologic diagnosis and death or last follow-up. Patients who were still alive at last follow-up were considered as a censored event. Progression-free survival (PFS) was defined as the time between histopathologic diagnosis and recurrence or last follow-up. Patients who were recurrence-free at last follow-up were considered as a censored event. In all analyses, a *p* value < 0.05 (two-sided) was considered statistically significant.

## SUPPLEMENTARY MATERIALS FIGURES AND TABLES


