# Supplementary material for: Whole genome duplication is an early event leading to aneuploidy in IDH-wild type glioblastoma
Source: Oncotarget. 2018 Nov 13;9(89):36017–28. doi: 10.18632/oncotarget.26330 (PMC6267593; doi:10.18632/oncotarget.26330)
Supplement: Supplementary file 1 [file oncotarget-09-36017-s001.pdf]

Whole genome duplication is an early event leading to aneuploidy in *IDH*-wild type glioblastoma

SUPPLEMENTARY MATERIALS

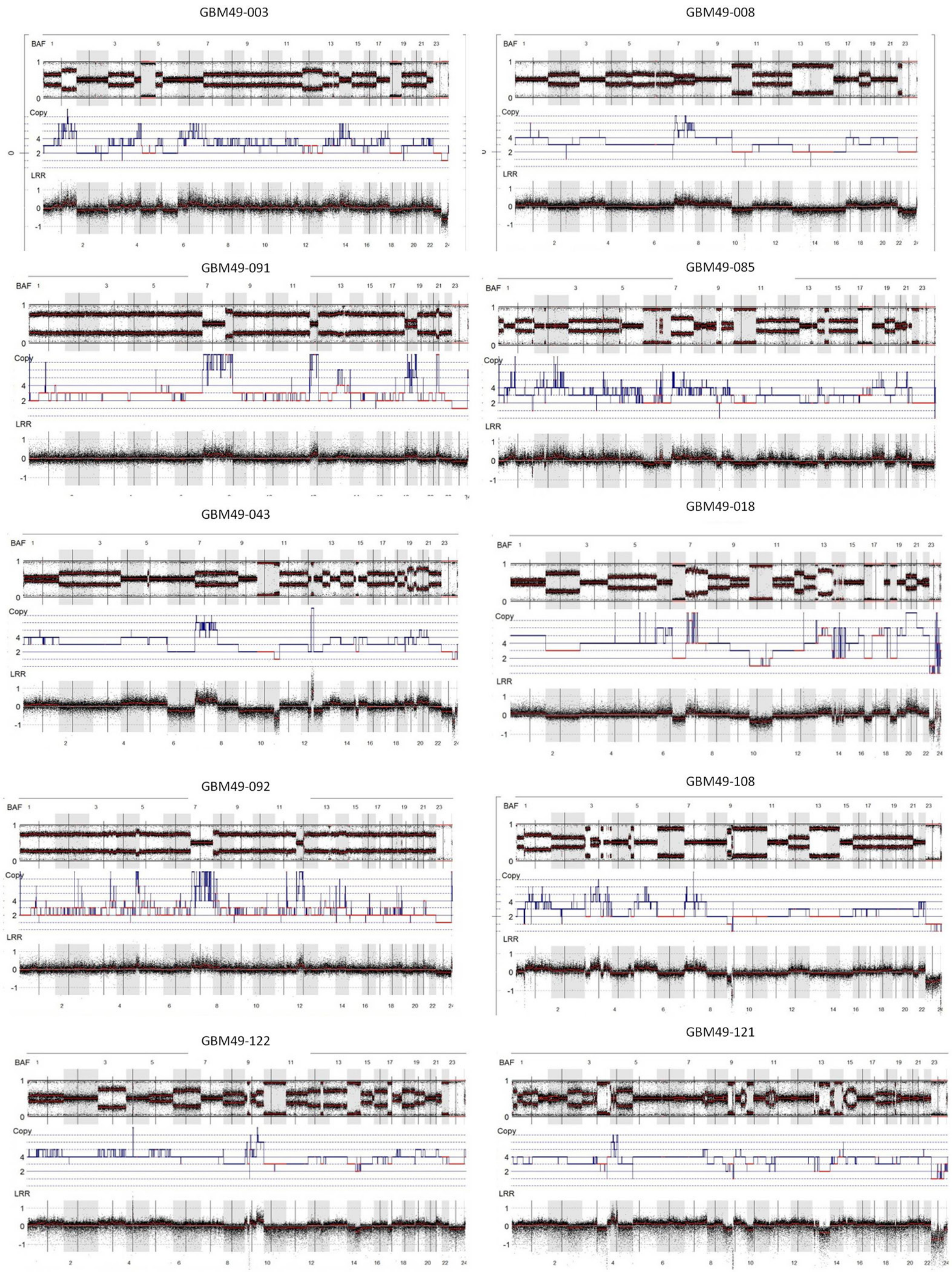

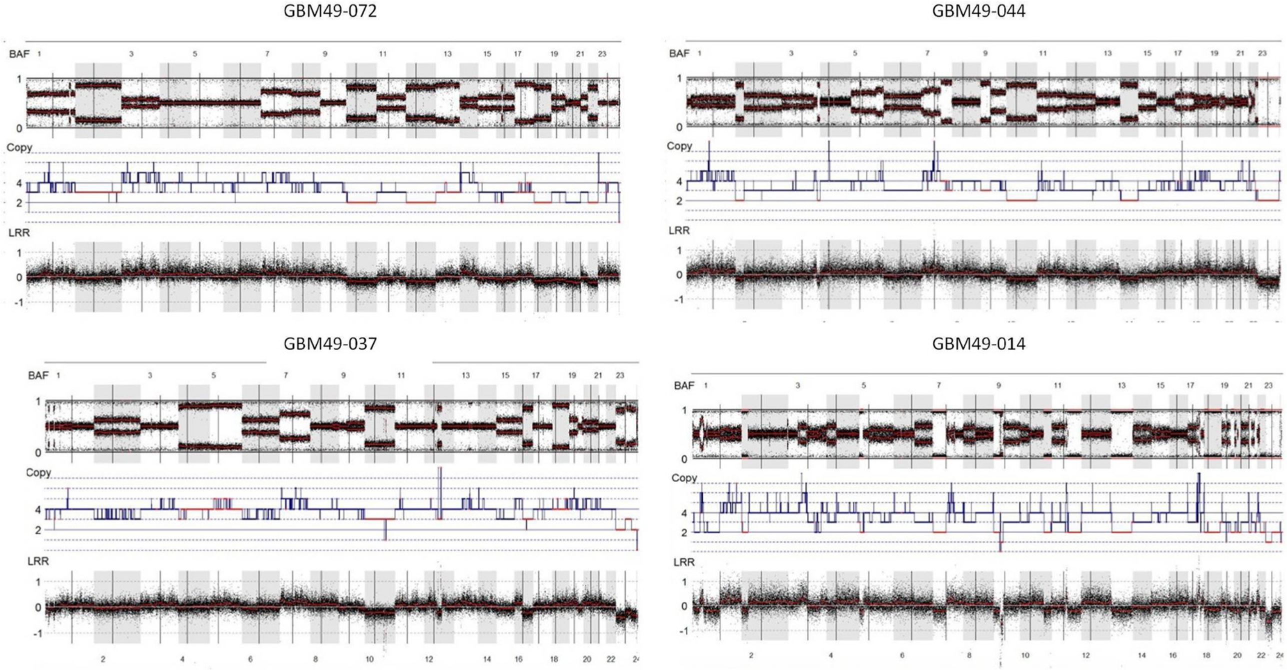

Supplementary Figure 1: The SNP array profiles were obtained by the GAP method[35].

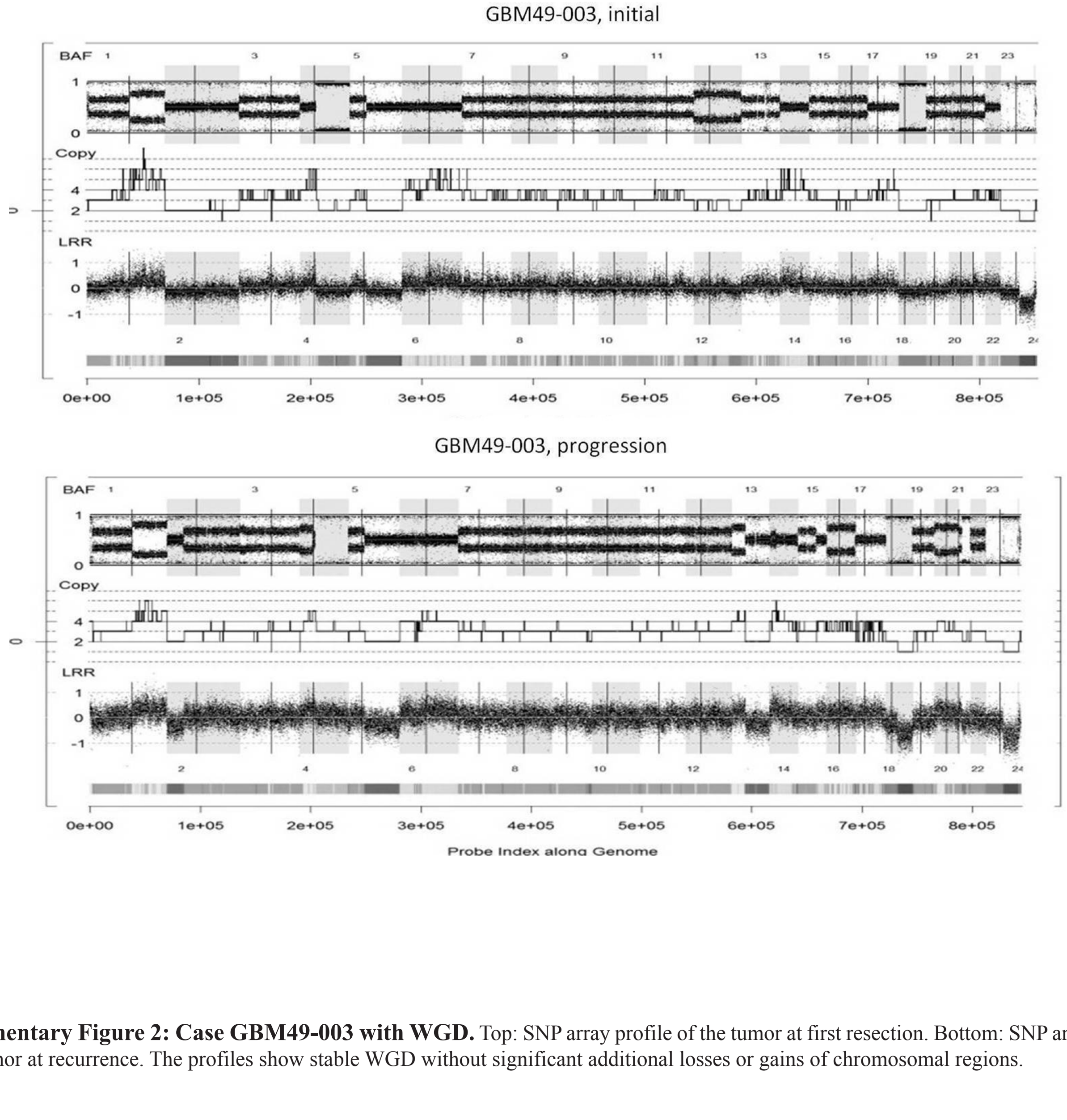

Supplementary Figure 2: Case GBM49-003 with WGD. Top: SNP array profile of the tumor at first resection. Bottom: SNP array profile of the tumor at recurrence. The profiles show stable WGD without significant additional losses or gains of chromosomal regions.

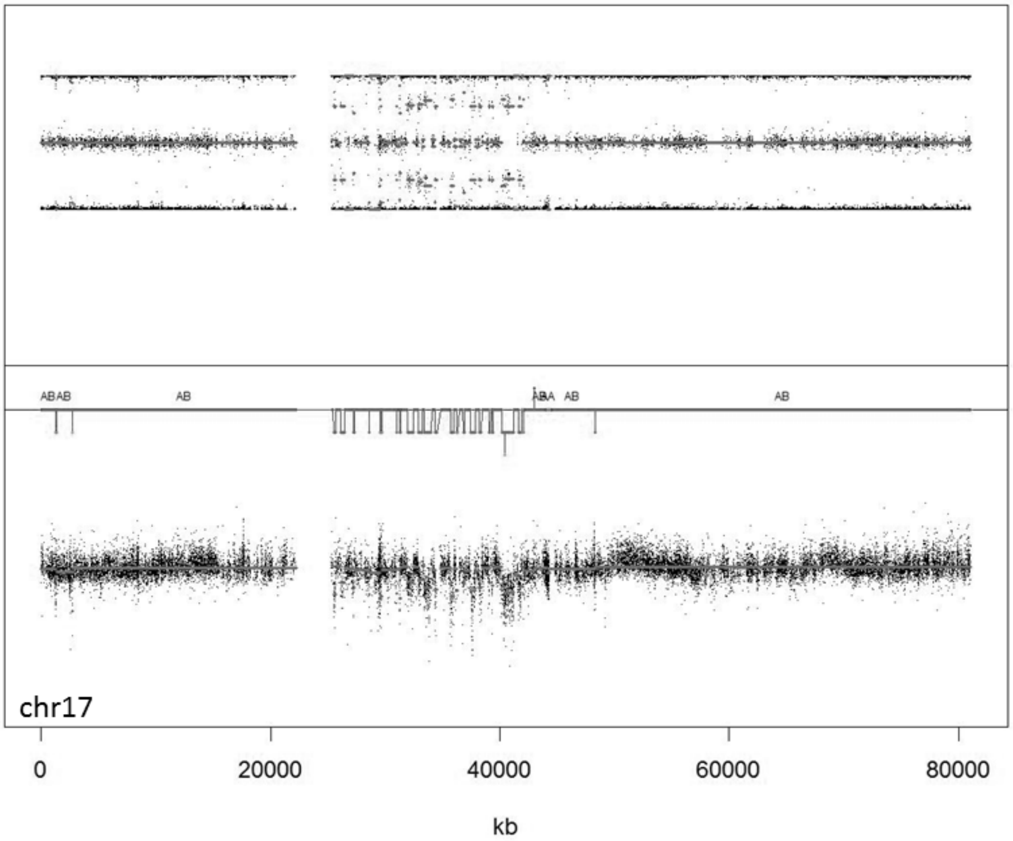

Supplementary Figure 3: GBM cases with an oscillating CT pattern on chr 17. CT also underlies oscillating rearrangements without homozygous deletion or gene amplification on chromosome 17.

Supplementary Table 1: List of primers for Sanger sequencing

Primers	Sequence 5′ to 3′
IDH1-Forward	AGAAGAGGGTTGAGGAGTTCAA
*IDH1*1-Reverse	ACACATACAAGTTGGAAATTTCTGG
IDH2-Forward	AGCCCATCATCTGCAAAAAC
IDH2-Reverse	CTAGGCGAGGAGCTCCAGT
TP53-ex2/3-Forward	AGAGAATGTGAAGCAGCC
TP53-ex4-Forward	TAAGGACAAGGGTTGGGC
TP53-ex5/6-Forward	TTCTTTGCTGCCGTCTTC
TP53-ex7-Forward	GCTGAGGAAGGAGAATGG
TP53-ex8/9-Forward	CTGGAGCTGGAGCTTAGG
TP53-ex10-Forward	TAGGTACTTGAAGTGCAGTTTC
TP53-ex11-Forward	GGGAAGATTACGAGACTAATAC
*TP53*3-ex2/3-Reverse	ATGGGTGAAAAGAGCAGTC
TP53-ex4-Reverse	TGACAGGAAGCCAAAGGG
TP53-ex5/6-Reverse	AACCCATTTACTTTGCACATC
TP53-ex7-Reverse	AGAAGCCACAGGTTAAGAG
TP53-ex8/9-Reverse	GGTGTTCTGAAGTTAGTTAGC
TP53-ex10-Reverse	CTGGGAGTTGCGGAGAATG
TP53-ex11-Reverse	TCTAAGCTGGTATGTCCTAC

Supplementary Table 2: FISH counting for WGD validation

		Number of signals detected per nucleus										
**ID sample**	**Chr**	**0**	**1**	**2**	**3**	**4**	**5**	**6**	**7**	**8**	**9**	**Average ploidy**
GBM49-001	3		3	31	41	18	6	1				2.96
	17	1	16	51	19	10	2	0	1			2.32
	8		4	21	28	23	11	7	5	1		3.62
GBM49-003	2		9	83	8							1.99
	16		3	34	57	4	2					2.68
	17		2	15	45	33	3	2				3.26
	19		4	32	55	7	2					2.71
GBM49-008	17		8	38	36	17	1					2.65
	18		2	70	28							2.26
	16	2	23	47	28							2.01
	10	10	32	52	4	2						1.56
	7		2	37	35	17	9					2.94
GBM49-018	1			13	24	27	19	10	6	1		4.11
	2		3	29	25	27	12	3		1		3.3
	3			10	27	27	27	9				3.98
	8		2	19	28	25	18	7	1			3.63
	9		3	29	43	20	4	1				2.96
	10	5	29	51	15							1.76
GBM49-091	17		44	44	10	2						1.7
	7			41	27	16	8	1	5		2	3.26
	18		45	34	13	8						1.84
GBM49-092	17		8	78	8	5	1					2.13
	7			20	32	22	11	8	5	2		3.78

Supplementary Table 3: Somatic copy number alterations in diploid *vs.* WGD GBM

	Diploid GBM	WGD GBM	*p* value
n	109	14	-
Median age	63 [22–84]	55 [23–79]	ns
Chr 9p loss	71 (65.1%)	7 (50%)	ns
Chr 7p gain	81 (74.3%)	8 (57.1%)	ns
Chr 10q loss	98 (89.9%)	8 (57.1%)	0.0044
Co-gain of chrs 19 and 20	28 (25.7%)	1 (7.1%)	ns
EGFR ampl	50 (45.9%)	3 (21.4%)	ns
CDKN2A HD	64 (58.7%)	7 (50.0%)	ns
CDK4 ampl	13 (11.9%)	3 (21.4%)	ns
MDM2 ampl	11 (10.1%)	2 (14.3%)	ns

Supplementary Table 4: Additional focal amplifications on chr 12 in *MDM2/CDK4* co-amplified cases

Sample ID	GBM49-022	GBM49-039	GBM49-030	GBM49-086	GBM49-119
Additional peak 1 location (Mb)	40.3–41.3	24.2–25.5	38.6 –42.9	56.7–56.8	96.0–100.2
Candidate gene 1	***LRRK2***, *CNTN1*	*SOX5, BCAT1, LRMP,* ***KRAS***, *CASC1*	12 genes including ***LRRK2***	***STAT2***	*CDK17, NEDD1*
Additional peak 2 location (Mb)	45.3–45.5	50.3–50.4		65.3–67.2	
Candidate gene 2	DBX2	RACGAP1		9 genes including, ***HMGA2***	
Additional peak 3 location (Mb)	74.9–75.8	60.8–61.5			
Candidate gene 3	*KCNC2, CASP2*	No gene			
